# Not heard or remembered: A daily assessment of experienced intersectional invisibility

**DOI:** 10.1111/bjso.70140

**Published:** 2026-10-11

**Authors:** Amanda K. Sesko, Béatrice Sternberg, Monica Biernat, Alexandra N. Fisher, Zaher Kmail

**Affiliations:** ^1^ Department of Social Sciences University of Washington Tacoma Tacoma Washington USA; ^2^ Department of Psychology University of Paris 8 Saint‐Denis France; ^3^ Department of Psychology University of Kansas Lawrence Kansas USA; ^4^ School of Philosophy, Psychology and Language Sciences University of Edinburgh Edinburgh UK; ^5^ Department of Sciences and Mathematics University of Washington Tacoma Tacoma Washington USA

**Keywords:** daily diary, health, intersectionality, invisibility, well‐being

## Abstract

Intersectional invisibility is the experience of not being remembered, attended to, differentiated, or given credit for contributions, and is most likely to be experienced by people with multiple stigmatized identities. In the present study, we used a smartphone‐based longitudinal daily diary design to examine the frequency and downstream consequences of experienced invisibility as they unfold in daily life. Over the course of 10 days, 301 participants reported their daily experiences of invisibility, sleep quality and positive affect, and pre‐ and post‐diary measures of well‐being and academic outcomes. As predicted, number of stigmatized identities positively predicted daily experiences of invisibility. For each additional stigmatized identity, the odds of reporting any instance of invisibility on any given day increased by 18%. Daily experienced invisibility was associated with poor outcomes, especially for those with multiple stigmatized identities, including reductions in daily positive affect and sleep quality, and pre‐ to post‐diary decreases in well‐being and academic outcomes. There was also evidence of cumulative effects on a short timescale: experienced invisibility from the previous 3 days predicted lower positive affect and worse sleep on the present day. This research suggests invisibility is a chronic experience with negative consequences, experienced by people with multiple stigmatized identities.

## INTRODUCTION

To me being invisible is when you are around people who you talk to, who know you but they don't notice you. It's like you are in their world as a side character and you aren't really able to be your own main character…you can go about your day without being noticed by anyone…at the store people don't look your way, at home you just kind of live there….That's what I feel like in most of my days. (Hispanic lower‐class first‐generation woman participant)

Invisibility is the experience of not being remembered, attended to, differentiated from others, or given credit for contributions (Fryberg & Townsend, 2008; Sesko & Biernat, 2010). In this research, we focus on *intersectional invisibility*, acknowledging that people with multiple stigmatized identities are most likely to experience these interpersonal slights (Purdie‐Vaughns & Eibach, 2008; Remedios & Snyder, 2018; Sesko & Biernat, 2025). Feminist scholars have detailed the role of intersecting marginalized identities (race, gender, class) in the socio‐political erasure and everyday exclusion of Black women (Combahee River Collective, 1995; Crenshaw, 1991; Davis, 1981; hooks, 1981; King, 1988). Psychological person perception research has documented invisibility effects through memory and social categorization errors made about targets who are members of two (versus zero or one) stigmatized groups. For example, Black women's faces are less likely to be remembered and their spoken statements are more likely to be misattributed than those of White women and Black and White men (Sesko & Biernat, 2010).

The current research focuses on experiences of invisibility among people with multiple, intersecting, stigmatized identities. Using a daily diary assessment approach, we aimed to better understand the association between number of stigmatized identities and everyday experiences of invisibility as well as downstream consequences for well‐being, health and academic outcomes.

### Theoretical frameworks and empirical evidence of intersectional invisibility

Intersectionality theory asserts that the multitude of social identities to which people belong interact in unique ways that influence everyday life (Beale, 1979; Crenshaw, 1991; King, 1988; Settles, 2006). Research that considers a single stigmatized identity in isolation (e.g., gender or race), overlooks experiences arising from the interplay with other identities (e.g., gender × race). Intersectional invisibility has been proposed as a marginalizing experience based on membership in multiple intersecting stigmatized identities (Purdie‐Vaughns & Eibach, 2008; Sesko & Biernat, 2010, 2025). Much quantitative research on intersectional invisibility in psychology has centred on how people are perceived by others; our research focuses on how people with multiple stigmatized identities *experience* invisibility in their daily lives.

#### Perceiver effects

Intersectional invisibility is theorized to occur because ethnocentrism, androcentrism, and heterocentrism contribute to the centring of straight, White men as the most prototypical society members, leaving others who belong to devalued social categories as less prototypical exemplars (Purdie‐Vaughns & Eibach, 2008). People with multiple stigmatized identities are more likely than those with a single stigmatized identity to be perceived as less prototypical members of their constituent groups. For instance, women of colour tend to share only a few stereotypes with their gender and racial/ethnic groups (Billups et al., 2022; Ghavami & Peplau, 2013). Similarly, gay Black men are judged to be less similar to the group ‘men’ than gay White men or straight Black and White men (Preddie & Biernat, 2021).

Intersectional invisibility effects have been operationalized in perceptual processes such as memory and social categorization errors (Lei et al., 2020; Schug et al., 2015; Sesko & Biernat, 2010, 2018; Sternberg et al., 2024). Sesko and Biernat (2010) defined invisibility as a lack of individuation or differentiation, evident in misattributions of spoken statements and poor face recognition of Black women compared to White women, Black men and White men. Others have found that participants are slower and less accurate in categorizing Black women's race and gender relative to White women and Black men (Goff et al., 2008; Johnson et al., 2012; Thomas et al., 2014; Zárate & Smith, 1990).

Several theories of intersectional person perception provide a framework for understanding the mechanisms that drive intersectional invisibility (for a review, see Sternberg et al., 2023). Johnson et al.'s (2012) *gendered race theory* asserts that race categorizations bias gender categorizations. For example, in the United States, the category ‘Black’ shares stereotypical attributes with the category ‘men’ rendering faster gender categorization for Black men than Black women (Johnson et al., 2012). Others theorize perceived *incongruence* between intersecting categories leads to perceptual errors, including invisibility effects (Rakić et al., 2020; Sternberg et al., 2024). Sternberg et al. (2024) found that participants made more memory errors for statements said by North African gay men (two minoritized identities in France) when they personally believed these two identities were highly incongruent. Participants who perceived Black women to be less prototypical of the group ‘women’ were more likely to commit errors in recognizing their faces (Sesko & Biernat, 2018).

Lei et al. (2023) suggest that how members of intersecting social categories are perceived is sociohistorically constructed, in that dominant gender and ethnic groups in a given context define prototypicality (cultural ideals) and thereby affect mental representations. These representations are learned early in development. For example, Lei et al. (2020) found evidence of intersectional invisibility effects in children: By age 5, children were slower to categorize Black women (compared to White and Asian women) by gender, were less likely to attribute feminine stereotypes to them, and were more likely to miscategorize them as men (also see Lei et al., 2022).

Deviation from prototypes or ideals—incongruency—seems to render certain groups ‘invisible’ to perceivers. To the extent that perceivers treat members of intersecting marginalized groups as invisible, these group members are likely to *feel* invisible in their daily lives. These feelings or experiences of invisibility have been described in a number of qualitative studies, but a quantitative psychological lens has been less frequently applied. This is the focus of our research.

#### From perceptions to experiences of invisibility

##### Stigmatized identities and feeling invisible

Research supports an association between intersecting stigmatized identities and experiences of invisibility. In Lewis and Neville's (2015) research on gendered racial microaggressions, 91% of Black women reported experiencing at least one invisibility‐related microaggression, such as feeling unheard or having their contributions ignored. A large body of qualitative research has further documented experiences of intersectional invisibility in contexts including healthcare settings (Okoro et al., 2022), higher education (Jackson et al., 2022; Wilkins‐Yel et al., 2019), and the workplace (Bhattacharyya & Berdahl, 2023; McCluney & Rabelo, 2019; Torbor et al., 2026).

Building on perceiver‐based research, we operationalize invisibility as everyday experiences of going ‘unnoticed’ and ‘unheard’. We propose that, as stigmatized identities accrue, people face greater risk of feeling invisible in their daily lives (Remedios & Snyder, 2018; Sesko & Biernat, 2025). This approach treats the accumulation of stigmatized identities additively, assuming that each additional stigmatized identity contributes comparably to the risk of invisibility. Additive approaches have been criticized for overlooking a central premise of intersectionality theory, which posits that identities interact to produce unique outcomes (Crenshaw, 1991; Warner, 2008). But others have noted that additive analyses can provide an informative step towards more fully interactive accounts (Bowleg, 2008). Showing that experiences of invisibility increase as stigmatized identities accumulate would establish whether members of multiple stigmatized groups face an elevated risk of being unnoticed or unheard, and can set the stage for research on how the nature and meaning of invisibility vary across specific intersections and social contexts.

Some existing evidence points to the association between number of stigmatized identities and experienced invisibility. Remedios and Snyder (2018) found that participants with two or three stigmatized identities reported feeling more invisible *because of their social identities* (race/ethnicity, gender, age, sexual orientation, social class), than those with zero or one stigmatized identity. This measure of invisibility explicitly mentioned social identities as the reason for the experience, and the association may be based on this measurement artefact. Sesko and Biernat (2025) measured experienced invisibility with a 13‐item index that made no reference to identities and tapped into feelings of being unnoticed and unheard, such as ‘Things I have said are sometimes misremembered as being said by someone else’ and ‘Sometimes people who should know me don't recognize me’. Across three samples, the number of stigmatized identities participants reported (a gender other than man, race other than White, sexual orientation other than heterosexual, etc.) positively predicted experienced invisibility. Invisibility was also correlated with, but distinct from, other measures of discrimination and invisibility‐related experiences, including hypervisibility (tokenism, heightened scrutiny) and strategic invisibility (intentionally reducing visibility to avoid scrutiny; Ballakrishnen et al., 2019; Settles et al., 2019). But this research was cross‐sectional in nature, offering no insight into longitudinal effects.

The experience of invisibility also resembles ostracism‐based social exclusion (where one is ignored in some way; Wesselmann et al., 2016; Williams, 2001). Ostracism is more likely to be reported by marginalized individuals (Büttner et al., 2025), and feeling ‘invisible’ is part of this experience (Williams, 2009). Studies of workplace and academic contexts have documented exclusionary experiences for Black women, including a lack of recognition for academic achievement (Settles et al., 2019), being excluded from conversations in campus spaces (Newton, 2023), being held to differential standards for evaluation, and feeling like an ‘outsider’ in workplace contexts (Bell & Nkomo, 2001; Berdahl & Moore, 2006; Smith et al., 2019).

While most people may occasionally experience exclusion (Neel & Lassetter, 2019), the meaning, frequency and consequences of such treatment are likely to vary based on afforded power and status (Cole, 2009; Fryberg & Eason, 2017). In this research, we argue that *intersectional* invisibility—experienced by those who are members of multiple stigmatized groups—is distinct from exclusion that may be occasional and/or less consequential (Remedios & Snyder, 2015; Sesko & Biernat, 2025); that is, less chronically oppressive (Pham et al., 2026). Therefore, we focus on number of stigmatized identities as a predictor of experienced invisibility and subsequent negative outcomes. Our focus is also intentionally narrow in that we draw on the person perception (perceiver) literature to define invisibility as feeling unseen and unheard. This approach increases measurement precision, reduces overlap with similar constructs, and affords specific analysis of the costs of these identified treatments for marginalized group members.

##### The costs of invisibility

Feeling invisible is associated with negative outcomes, including concerns about unfair treatment and being negatively stereotyped (Remedios & Snyder, 2018). Sesko and Biernat (2025) found that the number of stigmatized identities participants reported predicted experienced invisibility and lower well‐being, with invisibility mediating the relationship between number of stigmatized identities and poor well‐being. Invisibility may therefore be a form of minority stress (Meyer, 2003). In two of their three studies, Sesko and Biernat (2025) also found some evidence of a ‘social cure’ effect, whereby strong identification with one's groups served as a psychological resource that buffered the negative association between invisibility and well‐being (Haslam et al., 2018; Jetten et al., 2015), suggesting the effects of invisibility on outcomes can be tempered.

Identity‐specific stressors are harmful in the same manner as general stressors (Lazarus & Folkman, 1984; Meyer, 2003, 2007). Sleep is a physiological regulatory system (Irwin, 2002) that is responsive to stressors (Kim & Dimsdale, 2007) and predictive of poor health (Harvey, 2001; Muzni et al., 2020). Poor sleep has been identified as a mechanism predicting inflammatory burden in response to discrimination (Ong & Williams, 2019). To demonstrate the physiological toll of invisibility, our research evaluates sleep quality as a key health outcome. Minority stress predicts reductions to same‐day positive affect and increases to negative affect (Eldahan et al., 2016); we measured daily positive affect as an outcome potentially predicted by invisibility. Identity threats also reduce achievement and belonging (Cohen & Garcia, 2008; Walton & Cohen, 2011). Because our study focuses on college students, academic performance and belonging were also assessed.

### Daily life assessment of invisibility

Much research on experienced invisibility has been qualitative or cross‐sectional, but little is known about the frequency or consequences of day‐to‐day invisibility experiences as they unfold. In their correlational research, Sesko and Biernat (2025) found relatively low levels of experienced invisibility overall (mean ranges from 2.69 to 3.10, on a 7‐point rating scale). But invisibility is a complex experience to capture for several reasons. First, asking people to recall if they were misremembered assumes they have insight into others' perception errors. Second, asking people when or how often they were treated as invisible is subject to recall errors. A daily diary approach may provide more accurate estimates of invisibility experiences by reducing recall biases inherent in retrospective cross‐sectional measures. Sesko and Biernat (2025) also found evidence that experienced invisibility was associated with lower well‐being. But longitudinal data would be more effective in pointing to negative outcomes as a *consequence* of invisibility.

We used a smartphone‐based longitudinal daily assessment design to examine the frequency and consequences of experienced invisibility, thus providing an ecologically valid account of how invisibility unfolds in daily life. Daily life assessments using smartphone technology, often referred to broadly as Ecological Momentary Assessment (EMA) (or experience sampling methods [ESM], ambulatory assessment, intensive longitudinal data collection, daily diary), have become a popular method for capturing within‐person experience as it reduces limitations from lab‐based or traditional survey methods by (1) ensuring ecological validity, (2) reducing recall biases, and (3) increasing the reliability (relative to between‐person designs) of intra‐individual inferences made about the consequences of experiences (Koval et al., 2019; Trull & Ebner‐Priemer, 2014).

EMA designs have been used to capture the daily impact of exclusionary experiences similar to invisibility. Daily assessments have tracked affective responses to daily sexual objectification (Koval et al., 2019), the frequency of racial discrimination across contexts (English et al., 2020), and the consequences and coping strategies associated with daily ostracism (Bernstein et al., 2021; Kip et al., 2025; Pancani et al., 2023). EMA and ESM designs typically ask participants to respond to repeated surveys at random or set‐intervals throughout the day (measuring ‘in the moment’). In the current research, participants responded to end‐of‐day interval contingent prompts (retrospective to that day, fixed response time), best representing a ‘daily diary’ format.

### Overview of current research

Over 10 days, undergraduate students reported their daily experiences of invisibility and daily outcomes of sleep quality and positive affect. To capture changes in outcomes due to daily experienced invisibility, they completed pre‐ and post‐diary measures of well‐being, academic belonging and success. We therefore expand the array of outcomes that may be linked to invisibility. Following procedures by Sesko and Biernat (2025) general experiences of invisibility, discrimination experiences, hypervisibility and strategic invisibility were measured pre‐diary as covariates to distinguish the unique effects of invisibility.

#### Predictions

Predictions (H1–H7) were pre‐registered (https://aspredicted.org/bkrp‐938c.pdf).^1^ In H1, we expected general experienced invisibility measured pre‐diary to be positively correlated with daily experienced invisibility captured via the diary data.^2^

In our primary set of hypotheses, we predicted that: (H2) number of stigmatized identities would positively predict reported daily experiences of invisibility, (H3) on days participants experienced invisibility, they would also report lower sleep quality and less positive affect, (H4) daily experienced invisibility would negatively predict well‐being and academic outcomes (measured pre‐ to post‐diary), and (H5) average daily experienced invisibility would mediate the relationship between number of stigmatized identities and sleep quality, positive affect, academic outcomes and well‐being (Sesko & Biernat, 2025). In a subsidiary hypothesis (H6), we predicted the mediational effect in H5 would hold when controlling for related constructs, including other experiences of discrimination, strategic invisibility, hypervisibility and a single‐item identity‐based measure of invisibility.

In addition, we examined the ‘social cure’ hypothesis (Haslam et al., 2018): We predicted (H7) that high identification with one's social groups would buffer the negative relationships between invisibility and (a) sleep quality, (b) positive affect, (c) academic outcomes, and (d) well‐being.

## METHOD

### Participants

Participants were 301 undergraduates recruited from two distinct US universities: 227 students from a minority‐serving university (*M*_age_ = 22.52; *SD*_age_ = 6.90) and 74 from a majority White institution (*M*_age_ = 19.4; *SD*_age_ = 1.98), ensuring variability in the number of stigmatized identities. Data were analysed as combined samples. Tables 1 and 2 present demographic information. Sample 1 received course credit, a $20 gift card, and a $50 gift card lottery entry; Sample 2 received course credit.

**TABLE 1:** Sociodemographic characteristics of participants.

Characteristic	Sample 1 (*n* = 227)		Sample 2 (*n* = 74)		Total (*N* = 301)	
	*N*	%	*N*	%	*N*	%
Gender						
Women	141	62.4	34	52.3	175	60.1
Men	75	33.2	29	44.6	104	35.7
Non‐binary	8	3.5	1	1.5	9	3.1
Prefer to self‐describe	2	0.9	1	1.5	3	1.0
Race/ethnicity						
American Indian or Alaska Native	0	0.0	0	0.0	0	0.0
Asian	41	18.2	2	3.1	43	14.8
Black	33	14.7	3	4.6	36	12.4
Filipina/o/x	2	0.9	0	0.0	2	0.7
Latina/o/x/e	30	13.3	2	3.1	32	11.0
North African	2	0.9	0	0.0	2	0.7
Pacific Islander	7	3.1	0	0.0	7	2.4
White	67	29.8	48	73.8	115	39.7
Another race/ethnicity	5	2.2	0	0.0	5	1.7
More than one race/ethnicity	38	16.9	10	15.4	48	16.6
Sexual orientation						
Heterosexual	148	65.8	47	72.3	195	67.2
Asexual	8	3.6	4	6.2	12	4.1
Bisexual	30	13.3	9	13.8	39	13.4
Homosexual	15	6.7	1	1.5	16	5.5
Pansexual	5	2.2	1	1.5	6	2.1
Prefer to self‐describe	19	8.4	3	4.6	22	7.6
Social class^a^						
Lower class	54	23.9	3	4.6	57	19.6
Middle class	159	70.4	47	72.3	206	70.8
Upper class	13	5.8	15	23.1	28	9.6
International status						
Yes	4	1.8	1	1.5	5	1.7
No	222	98.2	64	98.5	286	98.3
English as primary language						
Yes	161	71.2	62	95.4	223	76.6
No	65	28.8	3	4.6	68	23.4

*Note*: Data were collected from May 2023–June 2025 for Sample 1 and March–May 2025 for Sample 2. Sample 2 was added to diversify the pool (and increase N) as Sample 1 included few participants with 0–1 stigmatized identities (also see Table 2). Percentages are based on available responses for each characteristic and may not sum to 100 due to rounding. International status = a temporary legal classification for foreign nationals studying in the United States.

^a^ On a 10‐point scale, the average subjective social class was 5.68 (*SD* = 1.66) in Sample 1 and 6.62 (*SD* = 1.51) in Sample 2.

**TABLE 2:** Number of stigmatized identities by sample.

Number of stigmatized identities	Sample 1 (*n* = 227)		Sample 2 (*n* = 74)		Total (*N* = 301)	
	*N*	%	*N*	%	*N*	%
0	14	6.2	15	23.1	29	9.9
1	22	9.7	27	41.5	49	16.8
2	58	25.6	11	16.9	69	23.6
3	61	26.9	8	12.3	69	23.6
4	48	21.1	3	4.6	51	17.5
5	22	9.7	0	0	22	7.5
6	2	.9	1	1.5	3	1.0
Intersectionality index^a^						
0–1 Stigmatized identities	36	15.9	42	64.6	78	26.7
2–6 Stigmatized identities	191	84.1	23	35.4	214	73.3

*Note*: Percentages are based on available responses for each characteristic and may not sum to 100 due to rounding. The intersectionality index is presented for descriptive reference, where 0–1 stigmatized identities represent those without multiple stigmatized identities and 2–6 represent those with multiple stigmatized identities.

^a^ The average number of stigmatized identities in Sample 1 was 2.80 (*SD* = 1.36), and in Sample 2 was 1.40 (*SD* = 1.25).

We aimed to collect as much data as possible within our financial and time constraints. Power analyses using Monte Carlo simulations estimating our central multilevel mediation analyses using observed effect sizes indicated the study is sufficiently powered (>80%).

Treatment of participants followed APA ethical standards and IRB approvals were received before data collection (Sample 1—University of Washington Tacoma: #STUDY00013233; Sample 2—University of Kansas: #STUDY00151862).

Data files, code, and materials can be found at: https://researchbox.org/4861.

### Materials and procedures

The study comprised three phases: (1) a pre‐diary survey, (2) a 10‐day daily diary assessment, and (3) a post‐diary survey. Participants reported to the lab for phase one, where they provided consent, completed the pre‐diary survey, and downloaded the Smartphone Ecological Momentary Assessment (SEMA3; O'Brien et al., 2024) application. For each of the following 10 days, participants received daily notifications through the SEMA3 app, at 7:30 pm, prompting them to complete a 5‐min survey measuring daily experiences of invisibility, positive affect and sleep quality. If not yet completed, participants were prompted again at 9:44 PM, with a survey closure at 11:59 PM. Research assistants checked in with participants on Days 2 and 7 to answer questions and encourage continued participation. Participants demonstrated high compliance, with 68.1% providing measurements on all 10 days and an average of 9.18 daily measurements out of 10 diaries (*SD* = 1.95; range 0–10). On Day 10, after being prompted to take the final diary, participants were emailed at 7:35 PM and provided a link to the post‐diary survey.

#### Pre‐diary measures

##### General experienced invisibility

*General experienced invisibility*, was measured using Sesko and Biernat's (2025) Experienced Invisibility Scale (EIS; 12 items^3^; e.g., ‘My contributions to group discussions often go unnoticed’; *α* = .87; 1 = *not at all true*, to 7 = *very true* scales).

##### Well‐being

*Well‐being* was assessed using the 54‐item Comprehensive Inventory of Thriving (Su et al., 2014); e.g., ‘I feel good most of the time’ (1 = *strongly disagree* to 5 = *strongly agree*); *α* = .95.

Participants rated their *physical health over the last 2 months* (1 = *poor* to 7 = *excellent*), and their *sleep quality during the past month* (1 = *very bad* to 5 = *very good*).

##### Academic measures

*Perceptions of academic ability* were measured with three items from the perspectives of (a) general others (‘People in general think my academic ability is…’), (b) close others (‘People in the best position to know think my academic ability is…’), and (c) self‐evaluations (‘I think my academic ability is…’), on 1 = *extremely low* to 7 = *extremely high* scales; *α* = .66 (Sinclair et al., 2006).

Participants reported their sense of *belonging* at their university, using 12 items adapted from Pietri et al. (2018); for example, ‘I belong at [X University]’, on 1 = *strongly disagree* to 7 = *strongly agree* scales; *α* = .86.

In addition to these subjective measures, participants provided objective performance measures including their *attendance* (‘In the last two weeks how many classes did you miss?’), current *GPA*, and *highest* and *lowest numerical grades* earned this quarter/semester.

##### Number of stigmatized identities

To measure number of stigmatized identities, we used an additive approach (count) (see Remedios & Snyder, 2018; Sesko & Biernat, 2025). One point was added (each) for identifying with a gender other than ‘male’, a race or ethnicity other than White/non‐Hispanic, a sexual orientation other than heterosexual, a social class rating of 4 or lower on the MacArthur scale of subjective social status (Adler et al., 2000), a first‐generation college student, an international student and a non‐native English speaker. Scores on this index could range from 0 to 7.

##### Group identification

As in Sesko and Biernat (2025), a holistic measure of group identification was used that captured felt connection to singular identities and the intersectional identity most studied in the invisibility literature (race × gender, reflecting the foundational focus on Black women's experiences; Aguayo‐Romero, 2021). Participants indicated how much they identified with each of their demographic groups (gender, race, age, social class, sexual orientation and race by gender; 1 = *do not identify at all with this group* to 7 = *identify very strongly with this group;* average of the six items; *α* = .83).

##### Invisibility covariates

To establish the distinct impacts of daily experienced invisibility, several covariates were measured (see Sesko & Biernat, 2025). Participants reported *experienced discrimination* based on their (1) ‘race, ethnicity, or color’, (2) ‘gender or gender identity’ (3) ‘sexual orientation’ and (4) ‘social class’; 1 = *never* to 7 = *all the time*; *α* = .70. *Fear of discrimination* was measured using five items adapted from Williams et al. (1997); for example, ‘You receive poorer service than other people at restaurants or stores’ on 1 = *not fearful at all* to 7 = *extremely fearful* scales; *α* = .87.

Less is known about hypervisibility and strategic invisibility; nonetheless, evidence suggests they co‐occur with invisibility, perhaps as a cluster of experiences tied to minoritized identities (Sesko & Biernat, 2025; Settles et al., 2019). S*trategic invisibility* was measured using five items (e.g., ‘Sometimes I find it useful to remain silent in group settings’; *α* = .73), and *hypervisibility* using six items (e.g., ‘There are times when I feel that things I do are scrutinized too closely by others’; *α* = .71; 1 = *not at all true*, to 7 = *very true* scales; Sesko & Biernat, 2025). *Identity‐based invisibility* and *identity‐based hypervisibility* were assessed using single items: ‘I feel invisible because of my identities (race/ethnicity, gender, age, sexual orientation, social class)’ (Remedios & Snyder, 2018) and ‘I feel hyper‐visible (excessively noticed and scrutinized) because of my identities (race/ethnicity, gender, age, sexual orientation, social class)’ (Sesko & Biernat, 2025), on 1 = *Strongly disagree* to 7 = *Strongly agree* scales.

#### Daily diary measures

##### Daily experienced invisibility

Daily experienced invisibility was measured using six items adapted from the 12‐item Experienced Invisibility Scale (Sesko & Biernat, 2025). Fewer items were chosen to reduce daily time burden and increase response rates. We chose the six most face‐valid items that were best adaptable to daily reports; participants indicated whether or not they had experienced each of the following events during that day: ‘My contribution to a group discussion went unnoticed’; ‘Something I said was misremembered as being said by someone else’; ‘Someone who should know me didn't recognize me’; ‘Someone confused me with someone else’, and ‘I was ignored’, responding categorically, *Yes* or *No* (‘count’ measure). The sixth item, ‘I felt invisible’, served as a subjective overall experience of invisibility, measured on a continuous scale from 1 = *not at all* to 7 = *very much* (‘continuous’ measure).

When participants gave a yes response to any event, or answered 5–7 on the continuous measure, they were prompted to report where they experienced the event, checking all that applied: school, work, everyday interactions with friends or family, or everyday interactions with strangers.

##### Daily positive affect

Participants were asked: ‘How much have you experienced feeling happy today?’ and ‘How much have you experienced feeling unhappy today?’ (1 = *not at all* to 7 = *all day*; Diener et al., 2009). These items were highly correlated across observations, *r* = −.64, *p* < .001; negative affect was reverse scored and averaged with positive affect to create a reliable measure of positive affect. Because we expect experiences of invisibility reduce happiness that day, relevant analyses predict day X positive affect from day X invisibility.

##### Daily sleep quality

Participants reported their subjective sleep quality, a health‐related outcome that can be measured daily and is associated with global sleep efficiency (Åkerstedt et al., 1994; Carney et al., 2012), by answering one item: ‘Last night, how would you rate your sleep quality?’ on a scale from 1 = *very bad* to 5 = *very good*. Fuller‐Rowell et al. (2021) found that experienced discrimination influences that night's sleep quality. We paired daily sleep quality measures with reported experiences of invisibility from the *previous day* (experiences of invisibility reported on day X correspond with sleep on day X, which was measured on day X + 1).

#### Post‐diary measures

The post‐diary survey included the same well‐being and academic measures as in the pre‐diary survey, except participants reported their *expected end of quarter/semester* GPA.

## RESULTS

### Analysis plan

Analyses were conducted in R (R Core Team, 2020) primarily using the lme4 package (Bates et al., 2015) for multilevel modelling. We pre‐registered a general analysis plan, indicating predictions would be tested primarily with multilevel models, while reserving flexibility for data‐driven analyses. Multilevel models account for the nested structure of daily diary data (daily observations nested within participants). For daily (Level 1) models, daily experienced invisibility was operationalized as both a count (out of five possible occurrences) and a continuous variable (single item ‘I felt invisible’), and decomposed into within‐person deviations (daily fluctuations around each participant's mean) and between‐person means (stable individual differences). This decomposition allowed us to distinguish day‐to‐day associations (within‐person) from person‐level differences (between‐person). All daily models included a random intercept for participant and controlled for linear day in study and number of stigmatized identities (grand‐mean centred) where appropriate. Models were estimated using linear mixed effects with restricted maximum likelihood (REML) estimation. For inference, we report fixed‐effect estimates (b) with 95% confidence intervals. For a detailed description of analyses for each hypothesis, see Supplementary Material.

### Descriptive statistics and measurement correlation

Participants completed up to 10 days of diary responses, yielding 3010 daily observations. Some daily reports were incomplete, leaving 2762 responses available for analysis. Daily experienced invisibility showed considerable within‐person variability across days (ICC = .47), as did sleep quality (ICC = .31) and positive affect (ICC = .43). On average, participants reported daily invisibility of *M* = 2.26 (*SD* = 1.59), when measured continuously, and *M* = .39 (*SD* = .76) when measured by daily count, with participants reporting any experience of invisibility on 27.40% of days. These experiences of invisibility were most likely to occur in the context of everyday interactions with friends and family (43.9% of the time), followed by at school (26.2%), at work (19.3%) and in interactions with strangers (10.6%).

Confirming H1, general experienced invisibility measured pre‐diary correlated positively and significantly with the average of daily experienced invisibility, when the latter was measured as a continuous variable, *r* = .57, 95% CI [.482, .639], *t*(291) = 11.71, *p* < .001, and as a count variable, *r* = .42, 95% CI [.320, .509], *t*(291) = 7.88, *p* < .001. Presented in Table 3, daily experienced invisibility was similarly positively correlated with all pre‐diary measures of invisibility‐related and discrimination constructs. These relationships were moderate and generally smaller than those with the general invisibility scale from which the daily invisibility measure was derived, suggesting some degree of distinction.

**TABLE 3:** Correlations between daily experienced invisibility and pre‐diary invisibility, discrimination and health.

Variable	*M*	*SD*	Daily experienced invisibility	
			Continuous	Count
General experienced invisibility	2.80	.95	.57***	.42***
Strategic invisibility	4.37	1.19	.41***	.31***
Hypervisibility	3.35	1.10	.39***	.33***
Identity‐based invisibility	2.30	1.50	.34***	.29***
Identity‐based hypervisibility	2.88	1.76	.26***	.22***
Discrimination	2.07	.95	.40***	.42***
Fear of discrimination	3.33	1.61	.29***	.26**
Physical health last 2 months	4.46	1.52	−.17**	−.22***
Sleep quality last month	2.82	1.10	−.29***	−.24***

*Note*: *M* and *SD* are used to represent mean and standard deviation, respectively.

*** *p* < .001.

** *p* < .01.

### Does number of stigmatized identities predict daily experiences of invisibility?

Consistent with H2, number of stigmatized identities positively predicted daily experiences of invisibility (measured continuously), *b* = .20, *SE* = .05, *t*(284) = 4.26, 95% CI [.105, .285], *p* < .001. Each additional stigmatized identity corresponded to a .19 unit increase in daily experienced invisibility. Based on this model, the estimated experienced invisibility score for someone with no stigmatized identities is 1.78 but rises to 2.94 for someone with six stigmatized identities.

Number of stigmatized identities also positively predicted daily count of experiences of invisibility, *b* = .16, *SE* = .05, *z* = 3.59, 95% CI [.074, .252], *p* < .001, IRR = 1.18. An IRR of 1.18 indicates that the mean expected count of invisibility experiences was 18% higher for every additional stigmatized identity.^4^

### On days participants experience greater invisibility, do they report lower sleep quality and less positive affect?

To test H3, we fit multilevel models predicting daily sleep quality and daily positive affect from within‐person daily invisibility (deviations around participant's own mean) and between‐person invisibility (participant means), controlling for day in study and number of stigmatized identities (grand‐mean centred).

On days when participants experienced more invisibility (continuous) than their own average, they reported significantly lower positive affect; see Table 4 (within‐person row). Unexpectedly, experiencing greater invisibility (continuous) than usual was linked to better sleep quality that same night (measured retrospectively the next day). However, participants with higher daily experienced invisibility on average reported lower sleep quality and lower positive affect (between‐person row).

**TABLE 4:** Multilevel models predicting same‐day affect and sleep from daily experienced invisibility (measured continuously).

	Daily affect					Daily sleep				
	*b*	*SE*	*t*	*df*	95% CI	*b*	*SE*	*t*	*df*	95% CI
*Fixed effects*										
Intercept	6.29***	0.11	58.60	282	[6.08, 6.51]	3.83***	0.10	38.36	274	[3.63, 4.03]
Daily experienced invisibility (continuous; within‐person)	−0.29***	0.02	−14.74	2228	[−0.33, −0.25]	0.05*	0.02	2.25	1838	[0.01, 0.09]
Daily experienced invisibility (continuous; between‐person)	−0.58***	0.04	−13.55	283	[−0.66, −0.49]	−0.22***	0.04	−5.44	277	[−0.29, −0.14]
Day in study (centred)	0.01	0.01	1.20	2261	[−0.01, 0.02]	−0.00	0.01	−0.40	1865	[−0.02, 0.01]
Number of stigmatized identities (centred)	−0.04	0.03	−1.26	283	[−0.11, 0.02]	−0.04	0.03	−1.20	273	[−0.10, 0.02]
*Random effects*										
*SD* (intercept for ID)	0.71					0.63				
*SD* (residual: observation)	1.08					1.03				
ICC	0.30					0.27				

Abbreviations: *b*, unstandardized coefficient; CI, 95% confidence interval; *SE*, standard error.

*** *p* < .001.

* *p* < .05.

The results were similar for the daily count measure of experienced invisibility. On days when participants experienced more invisibility (total count) than usual, they reported lower positive affect, but no different sleep quality; see Table 5 (within‐person row). However, participants with higher average invisibility reported lower sleep quality and lower positive affect (between‐person row).

**TABLE 5:** Multilevel models predicting same‐day affect and sleep from daily experienced invisibility (measured by count).

	Daily affect					Daily sleep				
	*b*	*SE*	*t*	*df*	95% CI	*b*	*SE*	*t*	*df*	95% CI
Fixed effects										
Intercept	5.33***	0.08	70.01	281	[5.18, 5.48]	3.50***	0.06	58.41	273	[3.38, 3.61]
Daily experienced invisibility (count; within‐person)	−0.25***	0.04	−6.64	2237	[−0.33, −0.18]	−0.02	0.04	−0.56	1910	[−0.10, 0.05]
Daily experienced invisibility (count; between‐person)	−0.85***	0.13	−6.51	280	[−1.10, −0.59]	−0.39***	0.10	−3.78	276	[−0.59, −0.18]
Day in study (centred)	0.01	0.01	1.00	2253	[−0.01, 0.02]	−0.01	0.01	−0.68	1911	[−0.02, 0.01]
Number of stigmatized identities (centred)	−0.09*	0.04	−2.33	282	[−0.17, −0.01]	−0.05	0.03	−1.73	273	[−0.12, 0.01]
*Random effects*										
*SD* (Intercept for ID)	0.88					0.65				
*SD* (Residual: Observation)	1.12					1.03				
ICC	0.38					0.28				

Abbreviations: *b*, unstandardized coefficient; CI, 95% confidence interval; *SE*, standard error.

*** *p* < .001.

** *p* < .01.

* *p* < .05.

We also examined the cumulative effects of invisibility (count) on sleep and positive affect. While there were no cumulative effects of daily experienced invisibility on sleep quality or positive affect over the full course of the study, there was a significant impact of the previous 3 days of experienced invisibility on that day's sleep quality, *b* = −.38, *SE* = .10, *t*(264) = −3.77, 95% CI [−.579, −.183], *p* < .001, and positive affect, *b* = −.69, *SE* = .13, *t*(274) = −5.15, 95% CI [−.950, −.426], *p* < .001.

Pre‐diary measures of physical health and sleep quality over recent months were negatively correlated with daily invisibility, suggesting participants who are subject to more daily invisibility have worse overall health; see Table 3.

### Does daily experienced invisibility negatively predict change in well‐being and academic outcomes?

Next, we examined whether mean daily experienced invisibility negatively predicted change in well‐being and academic outcomes. Consistent with H4, mean daily invisibility (continuous) predicted pre‐to‐post study decreases in well‐being, *b* = −.07, *SE* = .02, *t*(227) = −4.31, 95% CI [−.101, −.038], *p* = .001, belonging, *b* = −.10, *SE* = .03, *t*(224) = −3.06, 95% CI [−.158, −.034], *p* = .003, perceived academic ability, *b* = −.09, *SE* = .03, *t*(282) = −2.82, 95% CI [−.147, −.026], *p* = .005 and reported GPA, *b* = −.06, *SE* = .02, *t*(222) = −2.64, 95% CI [−.096, −.014], *p* = .009. No effects were found for pre‐to‐post study change in attendance (*p* = .947), lowest grade (*p* = .570), or highest reported grade (*p* = .310).

Mean daily invisibility (count) predicted decreases in well‐being, *b* = −.12, *SE* = .04, *t*(227) = −3.10, 95% CI [−.193, −.043], *p* = .002, and lowest reported grade, *b* = −.05, *SE* = .03, *t*(226) = −2.07, 95% CI [−.107, −.003], *p* = .040. No effects were found for pre‐to‐post study change in belonging (*p* = .073), GPA (*p* = .055), attendance (*p* = .832), perceived academic ability (*p* = .150), or highest reported grade (*p* = .547).

### Does daily experienced invisibility mediate the relationship between number of stigmatized identities and outcome measures?

To evaluate H5, we tested whether participants' average daily experienced invisibility mediated the association between number of stigmatized identities and (a) daily sleep quality, (b) daily positive affect and (c) pre‐ to post‐diary outcomes.

#### Mediation for daily outcomes

As above, number of stigmatized identities positively predicted average daily invisibility, and average daily invisibility in turn predicted poorer average sleep quality and lower average positive affect (*p*s < .001). The Monte Carlo estimated confidence intervals for the indirect effects (*a* × *b*) of sleep, *indirect effect* = −.04, 95% CI [−.064, −.018], and positive affect, *indirect effect* = −.11, 95% CI [−.168, −.058], did not include zero, suggesting a significant indirect effect of average daily experienced invisibility for both outcomes. The direct effect of number of stigmatized identities on sleep quality, *c′* = −.04, *SE* = .03, *t*(276) = −1.20, 95% CI [−.100, .020], *p* = .229, and on positive affect *c′* = −.04, *SE* = .04, *t*(283) = −1.26, 95% CI [−.110, .020], *p* = .210, controlling for daily invisibility was not significant. Invisibility accounted for roughly 53% of the relationship between number of stigmatized identities and changes in sleep and 73% of the relationship with changes in positive affect. See Figure 1.

**FIGURE 1:**
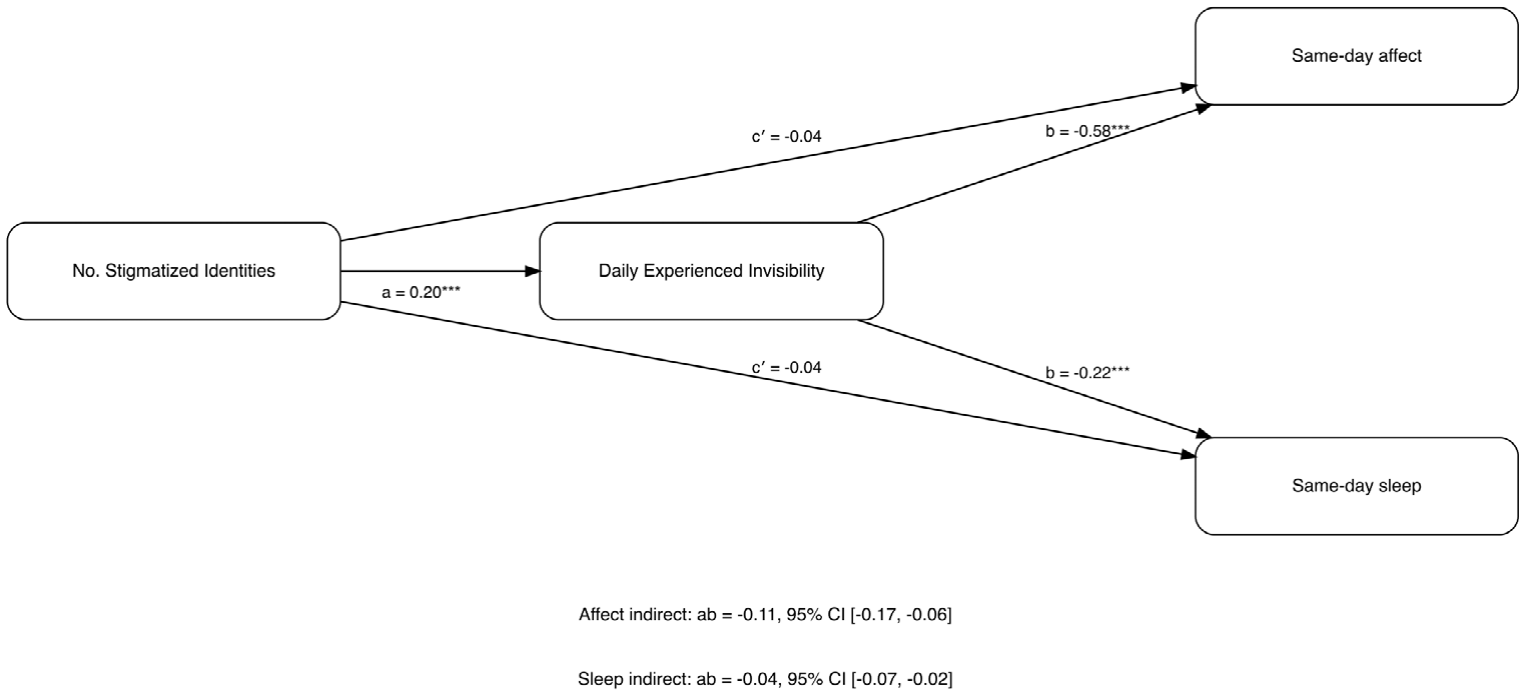
Mediation model: daily experienced invisibility mediates the relationship between number of stigmatized identities and same‐day sleep and positive affect. Paths are unstandardized coefficients; ***p < .001.

#### Mediation for pre‐ to post‐diary outcomes

Table 6 displays pre‐to‐post mediation results for well‐being and academic outcomes. Average daily experienced invisibility (measured continuously) mediated the association between number of stigmatized identities and pre‐to‐post decreases in well‐being, belonging and perceived academic ability. No other indirect effects were significant.

**TABLE 6:** Baseline‐adjusted mediation models (pre‐to‐post outcomes).

Outcome	*a* (X → M)	*p*	*b* (M → Y)	*p*	*c*′ (direct)	*p*	Indirect (*a* × *b*)	*p*	Total	*p*	Std. indirect
Well‐being	0.21 [0.111, 0.304]	<.001	−0.069 [−0.102, −0.038]	<.001	0.002 [−0.018, 0.021]	.846	−0.015 [−0.026, −0.007]	.002	−0.013 [−0.034, 0.009]	.243	−0.042
Belonging	0.207 [0.111, 0.304]	<.001	−0.096 [−0.164, −0.034]	.004	0.01 [−0.038, 0.056]	.674	−0.02 [−0.041, −0.007]	.018	−0.01 [−0.056, 0.036]	.673	−0.031
Perceived academic ability	0.193 [0.11, 0.275]	<.001	−0.087 [−0.153, −0.022]	.011	−0.034 [−0.083, 0.013]	.166	−0.017 [−0.035, −0.005]	.027	−0.051 [−0.102, −0.001]	.046	−0.029
GPA	0.217 [0.122, 0.315]	<.001	−0.055 [−0.137, 0.002]	.116	−0.002 [−0.028, 0.026]	.885	−0.012 [−0.037, 0]	.159	−0.014 [−0.04, 0.01]	.276	−0.038
Attendance	0.21 [0.111, 0.304]	<.001	−0.005 [−0.123, 0.134]	.941	−0.081 [−0.2, 0.024]	.154	−0.001 [−0.028, 0.028]	.943	−0.083 [−0.192, 0.019]	.124	−0.001
Lowest grade	0.196 [0.103, 0.294]	<.001	−0.006 [−0.029, 0.016]	.594	−0.016 [−0.034, −0.001]	.058	−0.001 [−0.006, 0.003]	.606	−0.017 [−0.034, −0.003]	.027	−0.007
Highest grade	0.207 [0.115, 0.305]	<.001	−0.003 [−0.009, 0.002]	.261	0.003 [−0.001, 0.009]	.254	−0.001 [−0.002, 0]	.292	0.002 [−0.002, 0.008]	.342	−0.014

*Note*: Cells show unstandardized *b* with 95% bootstrap CI in brackets. Indirect effects are *a* × *b*. *p*‐values are two‐tailed; mediation is supported when the indirect effect CI excludes 0. X = number of stigmatized identities, M = average daily experienced invisibility, Y = outcome.

#### Do the mediational results hold when controlling for related constructs?

To test H6, we added the following variables as covariates to our above (H5) mediation models: experiences of discrimination, strategic invisibility, hypervisibility, identity‐based invisibility and identity‐based hypervisibility. The indirect effect of the number of stigmatized identities on same‐day affect via daily experienced invisibility remained after controlling for related constructs, *indirect effec* *t* = −.10, 95% CI [−.146, −.049], as did the indirect effect for same‐day sleep quality, *indirect effect =* −.03, 95% CI [−.060, −.012].

We repeated the procedure for our mediation models for pre‐to‐post well‐being and academic outcomes. No pre‐post indirect effects remained after controlling for related constructs. See Figure 2 for a forest plot of indirect effects in models with and without controls.

**FIGURE 2:**
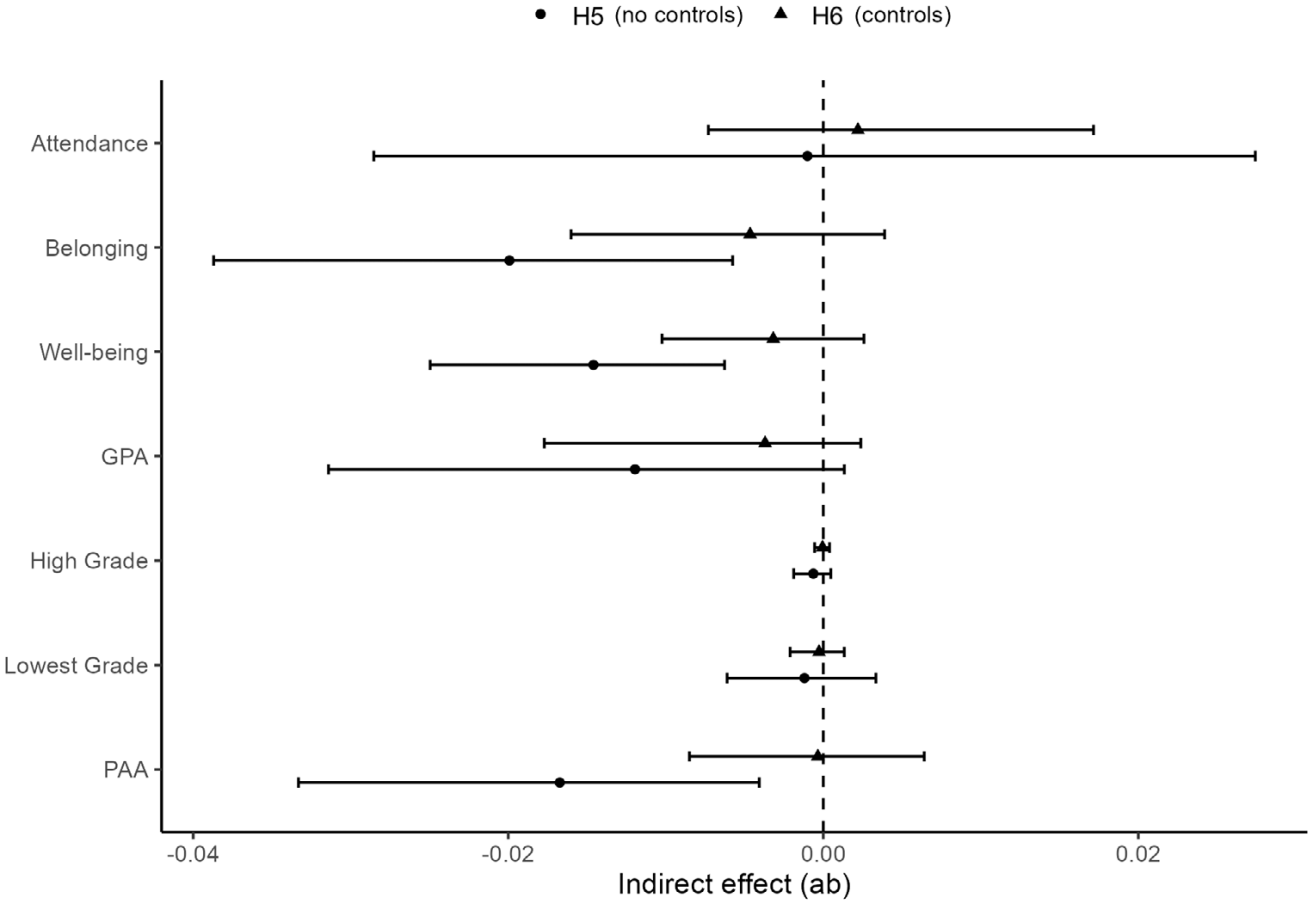
Comparison of indirect effects for pre‐post outcomes with and without controls. Horizontal lines represent 95% confidence intervals. H5, Hypothesis 5; H6, Hypothesis 6; PAA, perceived academic ability.

### Does group identification buffer the negative associations between experienced invisibility and outcome measures?

We examined whether group identification moderated the link between daily experienced invisibility and (a) sleep quality, (b) positive affect, (c) academic outcomes, and (d) well‐being (H7).

Group identification did not moderate the link between within‐person daily experienced invisibility (continuous) and sleep, *b* = −.01, *SE* = .02, *t*(1824) = −.60, 95% CI [−.048, .026], *p* = .552, nor between‐person daily experienced invisibility (continuous) and sleep, *b* = −.05, *SE* = .03, *t(*279) = −1.58, 95% CI [−.119, .013], *p* = .116. Nor did it moderate the link between within‐person experienced invisibility (continuous) and affect, *b* = .003, *SE* = .02, *t*(2219) = .160, 95% CI [−.033, .038], *p* = .873, or between‐person daily experienced invisibility (continuous) and affect, *b* = .04, *SE* = .04, *t*(281) =1.06, 95% CI [−.033, .109], *p* = .289. The results were the same when tested with count scores of daily experienced invisibility, all *p*s > .187.

Group identification did not buffer the negative associations between average daily experienced invisibility (continuous) and changes in well‐being, belonging, perceived academic ability, attendance, lowest grade or highest grade, all *p*s > .284. However, it did moderate the link between average daily experienced invisibility and pre‐to‐post change in GPA, *b* = .04, *SE* = .02, *t*(219) = 2.33, *p* = .021. Simple slopes analyses revealed that participants with lower group identification (1 *SD* below the mean) expected a decline in their GPA, *b* = −.10, *SE* = .03, *t*(219) = −3.37, *p* = .001, whereas participants with higher group identification (1 *SD* above the mean) did not, *b* = .00, *SE* = .03, *t*(219) = .01, *p* = .990.

The results were the same when tested with count scores of daily experienced invisibility: group identification only moderated the link between daily experienced invisibility and GPA, *b* = .10, *SE* = .05, 95% CI [.006, .196], *p* = .037. All other *p*s > .169.

## DISCUSSION

Our main goal was to understand how invisibility is experienced in daily life. On average, participants reported a greater than zero count of invisibility on 27.4% of days (i.e., roughly 3/10 days). Consistent with an intersectional invisibility account, participants with more stigmatized identities were most likely to report daily experiences of invisibility; for each additional stigmatized identity, the odds of reporting any instance of invisibility (count) on any given day increased by 18%. Given the nature of the daily assessment design, these data strongly support the notion that invisibility is chronically experienced by people with multiple stigmatized identities. These results replicate cross‐sectional correlational evidence (Sesko & Biernat, 2025) and demonstrate convergent validity across measurement types; daily invisibility was positively correlated with general experienced invisibility measured pre‐diary.

The hypothesis that daily experienced invisibility predicts poor outcomes was also supported. Experiencing greater daily invisibility (measured continuously and as a count) was associated with lower positive daily affect (that day and on average). Its association with sleep quality was more nuanced. At the between‐person level, higher average invisibility was associated with poorer sleep. At the within‐person level, however, sleep was unexpectedly better on days participants felt more invisible and not significant when measured as a count. While we suggest caution in interpreting these findings, we speculate they may indicate that feeling invisible is exhausting, increasing sleep on those nights. Daily counts of invisibility did not affect that night's sleep, but there was evidence of cumulative effects on a short timescale: experienced invisibility (counts) from the previous 3 days predicted lower positive affect and worse sleep on the present day, perhaps because of rumination that disrupted sleep (Reinka et al., 2020). However, cumulative effects did not extend across the full 10‐day study period, suggesting the impact of invisibility is temporally bounded.

Mediation analyses revealed the anticipated relationship with identity: Those with more stigmatized identities had more daily experiences of invisibility, which predicted both lower positive affect and poorer sleep quality. These effects remained when controlling for related constructs indicating that daily experienced invisibility played a unique role in the relationship between number of stigmatized identities and daily positive affect and sleep.

Daily experienced invisibility also predicted negative pre‐ to post‐diary changes in well‐being. For other outcomes, there was some discrepancy between the subjective rating and count variables: Decrements to perceived academic ability, belonging and GPA were associated only with subjective invisibility, and lowest grade reported with the incident count variable. Differences across these two continuous measures may reflect divergence between feeling invisible and reporting its specific occurrences. The count measure was more strongly right‐skewed than the subjective feeling measure, perhaps reducing its association with other variables. Further research is needed to assess the validity of these measures and reasons for their association with different outcomes.

Average daily experienced invisibility mediated the association between number of stigmatized identities and pre‐to‐post decreases in well‐being, belonging and perceived academic ability. However, these effects were less robust than for daily affect and sleep outcomes; after controlling for related constructs the indirect effects did not hold. Invisibility may be part of a cluster of oppressive experiences that simultaneously contribute to well‐being and other outcomes (Sesko & Biernat, 2025), and unique effects of invisibility as a mediator of the effect of number of stigmatized identities on some outcomes may not consistently emerge. Other variables not measured here (such as rumination or anticipated stigma, Reinka et al., 2020) may also moderate the effect of invisibility on outcomes, or the mediational patterns. Nonetheless, these results suggest that daily experienced invisibility has negative effects on a range of outcomes. Future research should continue to develop the scope of impacts for other psychological and performance outcomes (e.g., depression, chronic disease, promotions at work), and should consider alternative models. We did not examine whether daily experiences of positive affect and sleep affect later reports of invisibility, as such a causal order is not consistent with theory. A daily diary study cannot fully rule out mutual pathways, and the clearest test of the causal effect of invisibility will require experimental manipulations.

We did not find evidence of a ‘social cure’ effect: Identification with groups did not moderate the effect of daily experienced invisibility on negative outcomes, except for pre (current) to post (anticipated end of term) GPA. Daily experienced invisibility harmed perceptions of future academic success, but only for those without the protective barrier of high group identification. Sesko and Biernat (2025) found mixed evidence of this effect for well‐being, and failure to replicate it across most variables here suggests identification is an inconsistent buffer. Other research suggests identification is not always a protective mechanism–not all groups are good for us, and social groups are only a ‘cure’ to the extent that they offer support (see Jetten et al., 2014 for a review; McCoy & Major, 2003). A recent study found that for queer people of colour, invisibility was lower to the extent that frequent contact with ingroup members, and positive contact experiences with both ingroup and outgroup members occurred (Marei et al., 2026). Day‐to‐day experiences of invisibility may require immediate intervention to reduce its negative effects (e.g., sharing experiences with close others), which a general measure of identification does not capture. Future research should continue to establish the boundary conditions and replicability of identification as a buffer.

Daily assessments showed participants felt most invisible with friends and family, followed by at school, at work and with strangers. The higher occurrence with close others could be a byproduct of questions assuming acquaintance (‘Someone who should know me didn't recognize me’); yet, other questions used did not have this constraint. This pattern may merely reflect base rates of interactions, or invisibility may be noticed and felt more when the perceiver is known. Others have theorized invisibility is dynamic; participants report feeling invisible to some people and not to others (Lam & Neel, 2026). Future research should continue to investigate the impacts of invisibility across contexts and interpersonal relationships.

## CONCLUSION

Intersectionality theory suggests that the multitude of social identities to which people belong–not just single identities, in isolation–shape life experiences and outcomes. The current research further demonstrates that people with multiple stigmatized identities experience invisibility, daily and chronically, with negative consequences for well‐being and other outcomes. We hope for this research to aid scholars in identifying new ways to study invisibility and to mitigate its negative effects.

## AUTHOR CONTRIBUTIONS

**Amanda K. Sesko:** Conceptualization; methodology; data curation; investigation; funding acquisition; validation; visualization; formal analysis; project administration; supervision; resources; writing – original draft; writing – review and editing. **Béatrice Sternberg:** Conceptualization; methodology; data curation; investigation; validation; formal analysis; visualization; project administration; writing – original draft; writing – review and editing. **Monica Biernat:** Conceptualization; methodology; data curation; investigation; validation; supervision; project administration; resources; writing – review and editing; writing – original draft. **Alexandra N. Fisher:** Data curation; validation; formal analysis; writing – original draft; writing – review and editing; visualization. **Zaher Kmail:** Data curation; formal analysis; writing – review and editing; validation.

## CONFLICTS OF INTEREST

There are no competing interests to declare.

## ETHICS STATEMENT

Treatment of participants was in line with the ethical standards of the APA. Institutional review board (IRB) approval was obtained before collection of data (Sample 1—University of Washington‐IRB approval #STUDY00013233; Sample 2—University of Kansas‐IRB approval #STUDY00151862).

## Supporting information


**Data S1:** Supporting Information.

## Data Availability

The data that support the findings of this study are openly available at https://researchbox.org/4861.

## Footnotes

^1^
Pre‐registered predictions have been slightly reworded and organized for clarity.

^2^
We explored the role of identification in this relationship, asking if participants who felt strongly identified with their social groups would be more likely to report *daily* invisibility experiences rather than *general* experiences (evident in a lower correlation between general and daily invisibility). There was no evidence for this effect.

^3^
One item from the original 13‐item scale (“I feel that people take what I have to say seriously”) was removed based on psychometric results in a scale validation manuscript (Sesko et al., n.d.).

^4^
Sensitivity analyses excluding participants with zero or one stigmatized identity also supported H2; see Supporting Information.
