# Supplementary material for: Not heard or remembered: A daily assessment of experienced intersectional invisibility
Source: Br J Soc Psychol. 2026 Oct 11;65(4):e70140. doi: 10.1111/bjso.70140 (PMC13655358; doi:10.1111/bjso.70140)
Supplement: Supplementary file 1 [file BJSO-65-0-s001.docx]

**Supplementary Material** **for**

***Not heard or remembered: A daily assessment of experienced intersectional invisibility***

**Content Summary**

This document includes 1) a detailed description of the analysis plan for each hypothesis, and, 2) sensitivity analyses excluding participants with zero or one stigmatized identity for hypothesis 2 (H2).

**Analysis Plan by Hypothesis**

H1 assessed convergent validity by correlating the general (12-item) experienced invisibility scale with average daily experienced invisibility. H2 examined whether the number of stigmatized identities predicted daily experienced invisibility using mixed-effects models for continuous daily invisibility, and generalized mixed effect models with a Poisson distribution for count daily invisibility. H3 tested within-person associations between daily invisibility and same-day sleep quality and affect. For the models examining sleep quality, we created a lagged dataset to examine the effects of daily invisibility on that night’s sleep quality (measured retrospectively the following day). H4 examined whether participant’s average daily experienced invisibility predicted pre- to post-diary changes in well-being and academic outcomes using baseline-adjusted regression models (post-diary outcome regressed on average daily invisibility and the corresponding pre-diary outcome), controlling for number of stigmatized identities.

To evaluate process hypotheses, H5 used mediation models in which average daily experienced invisibility served as an indirect pathway linking number of stigmatized identities to daily outcomes and to pre-post outcomes. Indirect effects were estimated with confidence intervals matched to the model type. For daily mediation (stigmatized identities → mean daily invisibility → daily outcomes), indirect effects were computed as the product of relevant paths and evaluated using the Monte Carlo method for estimating confidence intervals based on 10,000 simulations of the a and b coefficients. Pre-post outcome mediation models were estimated in SEM using the lavaan package (Rosseel, 2012), and indirect effects were estimated using nonparametric bootstrap standard errors and confidence intervals with 10,000 bootstrap resamples. Results were interpreted as supporting a hypothesis when the 95% CI excluded zero for the focal effect (or when the incidence rate ratio (IRR) excluded 1 for count models). H6 assessed the robustness of the mediation by repeating mediation models while controlling for theoretically related constructs (e.g., fear of discrimination, strategic invisibility, hypervisibility, etc.) included as covariates in the outcome models.

H7 tested moderation by group identification by estimating invisibility x identification interactions for daily fluctuations and person-mean daily invisibility (moderation of both within- and between-person invisibility effects) on daily outcomes and group identification x average daily invisibility for pre-post outcomes.

**H2 Sensitivity Analyses**

Sensitivity analyses were conducted excluding participants with zero or one stigmatized identity (remaining *n* = 211). In the linear mixed-effects model (1,890 observations), number of stigmatized identities remained positively associated with daily experienced invisibility, *b* = 0.179, *SE* = 0.080, *t*(206.41) = 2.24, *p* = .026, 95% CI [0.022, 0.335].

The Poisson mixed-effects model of invisibility count (1,967 observations) yielded a consistent result. Number of stigmatized identities positively predicted the number of daily invisibility experiences, *b* = 0.162, *SE* = 0.071, *z* = 2.29, *p* = .022, IRR = 1.18, 95% CI [1.02, 1.35]. Thus, each additional stigmatized identity was associated with an estimated 17.6% increase in the expected daily invisibility count. Overall, the sensitivity analyses provided continued support for H2.

**References**

Rosseel, Y. (2012). lavaan: An R package for structural equation modeling. *Journal of*

*Statistical Software*, *48*(2), 1–36. https://doi.org/10.18637/jss.v048.i02
